# Breast cancer oestrogen independence mediated by *BCAR1 *or *BCAR3 *genes is transmitted through mechanisms distinct from the oestrogen receptor signalling pathway or the epidermal growth factor receptor signalling pathway

**DOI:** 10.1186/bcr954

**Published:** 2004-11-16

**Authors:** Lambert CJ Dorssers, Ton van Agthoven, Arend Brinkman, Jos Veldscholte, Marcel Smid, Koen J Dechering

**Affiliations:** 1 Department of Pathology, Josephine Nefkens Institute, Rotterdam, The Netherlands; 2 Target Discovery, N.V. Organon, Oss, The Netherlands

**Keywords:** anti-oestrogen, breast cancer, gene expression profiling, signal transduction, tamoxifen

## Abstract

**Introduction:**

Tamoxifen is effective for endocrine treatment of oestrogen receptor-positive breast cancers but ultimately fails due to the development of resistance. A functional screen in human breast cancer cells identified two *BCAR *genes causing oestrogen-independent proliferation. The *BCAR1 *and *BCAR3 *genes both encode components of intracellular signal transduction, but their direct effect on breast cancer cell proliferation is not known. The aim of this study was to investigate the growth control mediated by these *BCAR *genes by gene expression profiling.

**Methods:**

We have measured the expression changes induced by overexpression of the *BCAR1 *or *BCAR3 *gene in ZR-75-1 cells and have made direct comparisons with the expression changes after cell stimulation with oestrogen or epidermal growth factor (EGF). A comparison with published gene expression data of cell models and breast tumours is made.

**Results:**

Relatively few changes in gene expression were detected in the BCAR-transfected cells, in comparison with the extensive and distinct differences in gene expression induced by oestrogen or EGF. Both BCAR1 and BCAR3 regulate discrete sets of genes in these ZR-75-1-derived cells, indicating that the proliferation signalling proceeds along distinct pathways. Oestrogen-regulated genes in our cell model showed general concordance with reported data of cell models and gene expression association with oestrogen receptor status of breast tumours.

**Conclusions:**

The direct comparison of the expression profiles of BCAR transfectants and oestrogen or EGF-stimulated cells strongly suggests that anti-oestrogen-resistant cell proliferation is not caused by alternative activation of the oestrogen receptor or by the epidermal growth factor receptor signalling pathway.

## Introduction

The development and progression of breast cancer is dependent on steroid sex hormones and polypeptide growth factors. Oestrogen action has been implicated in the development of breast cancers and frequently contributes to tumour growth. The action of oestrogen is relayed through its specific nuclear oestrogen receptor (ER), which belongs to the family of ligand-inducible transcription factors [1,2]. Two distinct genes for ER (*ERα *and *ERβ*) have been identified. The role of ERα in breast cancer has been studied extensively and this receptor has been the subject of targeted therapies. Less information is available for ERβ [3,4], which exhibits differential tissue distribution and alternative responses to selective ER modulators [5,6]. Epidermal growth factor receptor (EGFR) expression is mainly present in ERα-negative breast tumours and is a marker of poor prognosis [7,8].

The frequent occurrence of ERα in breast tumours (about 75%) has been used as a guide for treatment. Endocrine treatment regimens, which either reduce the endogenous oestrogen levels (for example aromatase inhibitors) or interfere with ERα activation (anti-oestrogen such as tamoxifen), have been shown to block tumour growth and in some cases cause tumour reduction or disappearance [9,10]. However, the resistance of ERα-positive breast tumours is a severe limitation of endocrine treatment. About half of the ERα-positive breast tumours completely fail to respond (intrinsic resistance), whereas all responsive breast cancers ultimately progress and become resistant to the treatment (acquired resistance).

Despite much effort, the basis for the resistance of breast cancer to endocrine treatments is still poorly understood [11]. In general, tamoxifen resistance is not accompanied by loss of ERα expression [12,13]. Previous work has shown that the treatment outcome might be the result of a delicate balance between positive and negative regulators acting in concert with the hormone receptor [2,14]. In addition, alternative growth regulatory pathways might be available to tumour cells, permitting escape from the treatment [15]. We have searched for specific Breast Cancer Anti-oestrogen Resistance (*BCAR*) genes involved in the progression of oestrogen-dependent breast cancer cells to anti-oestrogen resistance [16]. The *BCAR3 *gene was shown to control anti-oestrogen-resistant cell growth in two different oestrogen-dependent cell lines and its product exhibits features of a cytoplasmic signalling molecule [17]. The *BCAR1 *gene causes anti-oestrogen resistance in our cell model and is the human homologue of the rat Crk-associated substrate (*p130Cas*) gene [18]. This docking protein has been implicated in many types of intracellular signalling processes [19,20]. Moreover, studies of human breast cancer specimens have shown that high BCAR1 expression is associated with poor prognosis and also predicts a poor response of recurrent disease to treatment with tamoxifen [21-24].

Recent developments in global gene expression profiling have elegantly shown their applicability in tumour classification and in predicting the prognosis of the patient [25-30]. Furthermore, studies in model systems have highlighted the use of gene expression profiling for unravelling delicate cellular processes.

The aim of our study was to use gene expression profiling to investigate the anti-oestrogen-resistant growth regulatory process induced by overexpression of the *BCAR *genes and to establish whether oestrogen or epidermal growth factor (EGF) signalling are involved.

## Materials and methods

### Breast cancer cell lines cultures and RNA preparation

The oestrogen-dependent human breast cancer cell line ZR-75-1 was maintained in RPMI 1640 medium supplemented with 10% bovine calf serum and 1 nM 17 β-oestradiol (R/BCS/E2) as described previously [16]. The derived EGFR-transfectant cell line ZR/HERc(1A) [31], hereafter referred to as ZR/EGFR, a BCAR1-transfectant cell line (4A12) [18] and a BCAR3-transfectant cell line (B3-10) [17] were also maintained in R/BCS/E2 medium. For short-term induction experiments, cells were cultured for 4 days in regular medium lacking added oestrogen (R/BCS) in 162 cm^2 ^flasks, given fresh R/BCS medium 24 hours before manipulation, and cultured for 6 hours in the presence of 100 nM oestradiol or 1 μM ICI 164,384 (or ethanol vehicle alone) in R/BCS medium. Hormones and anti-hormones were provided by N.V. Organon (Oss, The Netherlands). For long-term induction experiments, cells were grown for 7 days in R/BCS/E2 medium or R/BCS medium containing 10 ng/ml EGF (Roche Diagnostics Nederland B.V., Almere, The Netherlands). Medium was replaced after 3 days and at 24 hours before harvest. After completion of the culture, the medium was removed and cells were lysed directly with 16–20 ml of RNAzol B solution (Campro Scientific, Veenendaal, the Netherlands). RNA was prepared as described by the manufacturer, quantified and checked for integrity on agarose gels. Poly(A)^+ ^mRNA was prepared from pooled total RNA samples of two independent cultures by two cycles of binding to oligo(dT) with the use of OligoTex (Qiagen/Westburg B.V., Leusden, The Netherlands), and checked for integrity and contamination with ribosomal RNA by capillary electrophoresis (Lab-on-a-Chip, Agilent Technologies 2100 bioanalyzer, Amstelveen, The Netherlands).

### Expression analysis

Production of Cy5-labelled and Cy3-labelled cDNA from the purified mRNA, hybridisation of the UniGEMV cDNA microarrays and quantification of the signals were performed by Incyte Genomics (Mountain View, CA, USA) as described previously [32]. Two batches of UniGEMV2 microarrays were used for these experiments. All hybridisations (Table 1) were performed in duplicate with a fluor reversal to minimise possible bias caused by the molecular structure of the Cy3 and Cy5 dyes. Data analysis was performed with the Rosetta Resolver software package (v 3.2) with an Incyte/UniGEM microarray error model (Rosetta Inpharmatics Inc., Kirkland, WA, USA). Genes exhibiting at least once a significant difference (*P *≤ 0.01) in expression in these experiments were used for further analysis (*n *= 2373). In addition to the actual measured gene expression ratios we calculated the expression ratios between different experimental conditions from two measurements that contained a common sample [33]. Because the calculated gene expression ratios were in good agreement with available actual measurements, we used these calculations as 'virtual experiments'.

For hierarchical clustering of the measured and calculated expression ratios, we used Resolver software (average linkage agglomerative clustering using Euclidean distance and weighted by error) and Spotfire Decision Site 7.1 analysis package (Spotfire Inc., Somerville, MA, USA) using the Unweighted Paired-Group Method with Arithmetic mean (UPGMA) and Pearson's correlation as a similarity measure. Information on the function of genes has been retrieved from various public databases (for example PubMed, OMIN, GENECARD, KEGG and GO) and from the LifeSeq Gold database (Incyte Genomics). Expression data publicly available from prostate cancer, breast cancer and cell lines were linked to our data by means of the Unigene cluster number.

The strength of the relations between oestrogen and EGF-induced gene expression (log [ratio]) data was tested by Spearman rank correlation. The relations between categorised expression data (differential expression [DE] ≥ 1.60; 1.60 > DE > – 1.60; DE ≤ – 1.60) and gene association with tumour ER status (positive or negative) were tested by Spearman rank correlation. All computations were done with the STATA statistical package, release 8.0 (STATA Corp., College Station, TX, USA). All *P *values are two-sided.

### Quantitative RT–PCR

For quantitative reverse transcriptase-mediated polymerase chain reaction (RT–PCR), 2.5 μg of total RNA (or 100 ng of poly(A)^+ ^mRNA), 0.8 μg of oligo(dT)_12–18 _(Invitrogen Corporation) and 0.5 μg of random hexamer (Pharmacia) in 20 μl of RNase-free water were heated for 5 min at 65°C and cooled on ice. The final reaction of 40 μl contained 0.4 mM dNTPs (Pharmacia), 60 units of RNAseOUT and 300 units of SuperscriptII Reverse Transcriptase (Invitrogen). Incubations were for 2 min at 0°C, 10 min at 25°C, 50 min at 42°C and 10 min at 55°C; the reaction was stopped by heating for 15 min at 75°C. RNA was destroyed by treatment with 2 units of RNAseH (Promega) for 30 min at 37°C. cDNA products were diluted to 100 μl with 10 mM Tris/HCl pH 7.5; these stocks were stored at -80°C. Forty cycles of amplification of 5 μl of cDNA stocks in distilled water (Invitrogen) diluted 1:19 were performed with an SYBR green PCR mix (Applied Biosystems or Stratagene) and 0.33 μM forward and reverse primers in a volume of 25 μl on a ABI Prism 7700 (Applied Biosystems) in accordance with the recommended protocol. Primer annealing was performed at 60 or 62°C. A dilution series (1:4 to about 1:10,000) of a reference cDNA pool (mixture of cDNA preparations of RNA derived from six different cell lines) was used for normalising gene expression. The intron-spanning gene primers used are listed in Table 2. The cycling conditions were as follows: denaturation for 10 min at 95°C; 40 cycles (15 s at 95°C, 30 s at 60°C (CTSD, TFF1 and MYC) or 62°C, 10 s ramping to 72°C, 20 s at 72°C, 10 s ramping to 79°C, 20 s at 79°C). Data were collected at 72 and 79°C and were analysed at 79°C. At the end of the amplification, the melting curve of the products was determined. PCR products showed discrete melting curves and specific bands of correct lengths on agarose gels after 40 cycles of amplification. We also used Assays-on-Demand™ (Applied Biosystems) for various genes, in accordance with the manufacturer's protocol. cDNA (5 μl, diluted 1:19 or 1:39) was measured in 25 μl reactions with the TaqMan Universal PCR master Mix. All cDNA samples were normalised for *HPRT1 *levels (four independent measurements) and are presented relative to the gene level in ZR-75-1 cells maintained in R/BCS medium.

## Results

### Overall gene expression

To evaluate the effects of oestrogen, of EGF and of previously identified *BCAR *genes involved in oestrogen-independent growth of the human breast cancer cell line ZR-75-1, we determined the global gene expression in these cells by using UniGEMV2 cDNA microarrays. We performed direct comparisons of mRNA samples without the use of a general reference RNA sample (Table 1). Of the approximately 9000 sequences (about 88% represent known genes according to UNIGENE build no. 160) present on the microarray, the expression of 2373 genes (i.e. 26% of total sequences) was significantly (*P *≤ 0.01) affected by either the oestrogen treatment or the EGF treatment or the BCAR transfections. Hierarchical clustering distributes the expression profiles according to the experimental culture conditions; that is, profiles of long-term oestrogen-treated cells were separated from short-term oestrogen-treated cells or BCAR-transfected cells (Additional file 1). The majority of the large changes in gene expression were observed in the oestrogen-stimulated or EGF-stimulated cultures. Hybridisation profiles of short-term oestrogen stimulation of ZR-75-1 cells and BCAR3-transfected cells are very similar and distinct from hybridisation profiles of long-term oestrogen-stimulated cell lines (Additional file 1). The EGF-stimulated ZR/EGFR cells as well as the BCAR1- and BCAR3-transfected cells exhibit clearly different profiles in comparison with the oestrogen-stimulated cultures. The comparison of BCAR-transfected cell lines with each other or with non-stimulated parental cells revealed modest changes in gene expression, indicating that gene expression differences in these BCAR cell lines are generally subtle. Below we discuss the expression profiles (average gene expression log(ratios) of the independent experiments) of a selection of 1006 genes exhibiting a |DE| ≥ 1.60 in at least one of the actual or virtual experiments.

### Effects of oestrogen and EGF on gene expression of ZR-75-1 human breast cancer cells

**Figure 1:**
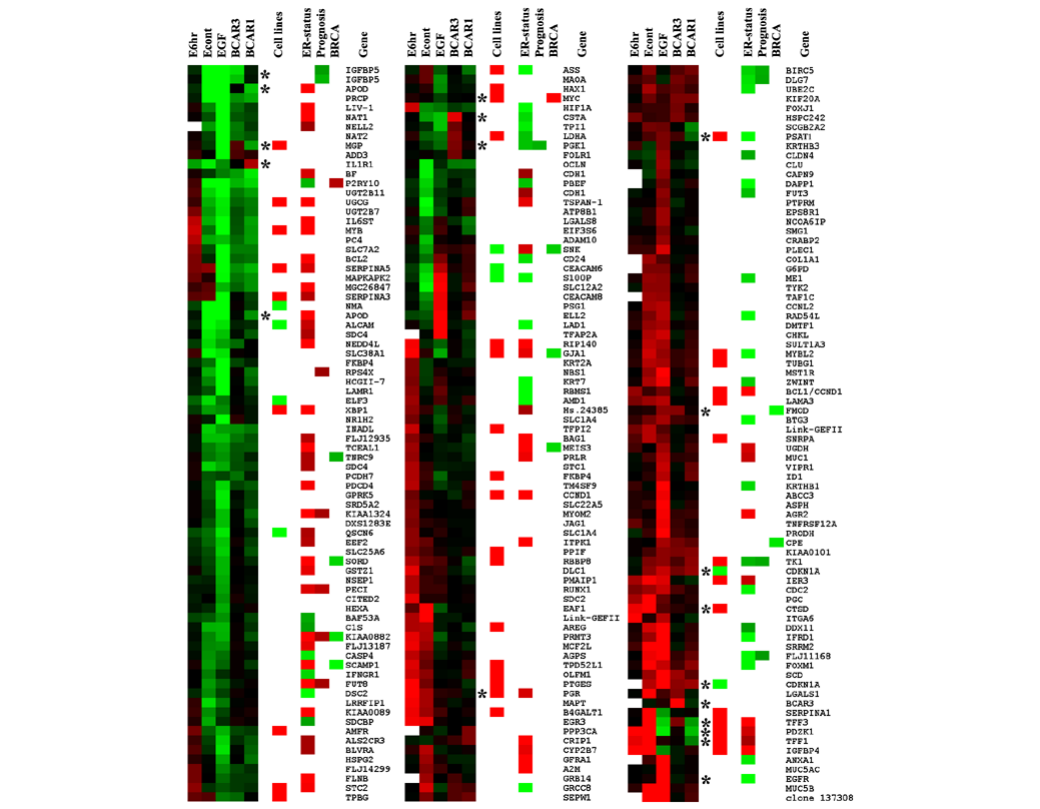
Expression changes of selected genes in ZR-75-1-derived cell lines, and their relation to reported clinical phenotypes. Average gene expression ratios (log_10_) in ZR-75-1 cells stimulated with oestrogen for 6 hours (E6hr) or continuously (Econt), of ZR/EGFR cells stimulated with epidermal growth factor for 7 days (EGF), of BCAR1-transfected or BCAR3-transfected ZR-75-1 cells versus unstimulated ZR-75-1 cells are indicated. A selection of 234 sequences was made from the 1006 sequences showing at least once a *P *value of 0.01 or less and exhibiting at least once a |DE| of more than 1.60. Individual data points of combined swapped and of virtual experiments were only included when either one of the following criteria was met: *P *< 0.051 or log(error) < 0.1761 or |log(ratio)| > 1.5 × log(error). This procedure eliminates most of the unreliable data points. Genes were hierarchically clustered by using Spotfire. A colour picture was made with TreeView [44]. Increased expression is shown in red and decreased expression is shown in green. Black represents no change and white indicates missing data. In addition, the reported inducing (red) or reducing (green) effects of oestrogen stimulation in cell line models [35–37,39,40] and the correlation (red, positive; green, negative) of individual genes with breast cancer oestrogen receptor (ER) status, *BRCA *mutation status (BRCA) and prognosis of disease recurrence is indicated [28,41]. Genes investigated with quantitative reverse transcriptase-mediated polymerase chain reaction have been marked with an asterisk. The complete list of 1006 genes with attached information is presented in Additional file 3.

ZR-75-1 breast cancer cells are completely dependent on oestrogen for growth. In standard medium without added oestrogen, growth is strongly reduced. Addition of anti-oestrogen completely abolishes the growth of these cells [16]. Oestrogen-induced cell proliferation is mediated by the transcription activation function of the ERα. To identify the early effects (that is, the transcription targets) of oestrogen stimulation, the expression profile was analysed after a 6-hour high-dose pulse of 100 nM oestrogen. From Fig. 1 and Additional file 1 it is clear that limited changes in gene expression (115 genes with |DE| ≥ 1.60) have occurred during this short treatment compared with mock-stimulated cultures. Over 75% of these genes seemed to be induced. Among these genes are well-known oestrogen targets such as *TFF1 *(PS2), *CSTD *(cathepsin D), *CCND1 *(cyclin D1) and *PGR *(progesterone receptor). These and several novel genes were rapidly induced by oestrogen both in the parental ZR-75-1 cells and in the BCAR3-transfected cells (Fig. 1 and Additional files 1,2,3). An extended picture emerges after continuous exposure to the regular dose of 1 nM oestrogen. About 400 genes exhibit consistent changes (at least 1.6-fold) in expression, of which about 60% of the sequences exhibit a significant decrease of gene expression (up to sevenfold) and 40% are increased (up to more than 10-fold). The genes specifically modulated by oestrogen comprise members of all functional compartments and processes in the cell. One-quarter of the early-induced genes remain expressed (DE > 1.60) during continuous exposure to oestrogen (see Fig. 1), whereas the expression of others is turned off (for example *AMD1*, *BCL2*, *CCND1*, *GJA1*, *MEIS3*, *RIP140*, *RUNX1 *and *STC1*) or even downregulated (for example *HIF1A*, *IL6ST*, *MYB*, *PC4 *and *UGT2B7*). Definitions of these and other and other gene names can be found in Additional file 3. Statistical analysis of all 1006 genes shows a clear positive correlation between early-induced gene expression and genes expressed after 7 days of oestrogen treatment (*r*_s _= 0.36, *P *< 0.0001).

We have previously shown that the addition of EGF to the culture medium does not support growth of ZR-75-1 cells because of the absence of EGF receptors [31,34]. The introduction of *EGFR *into ZR-75-1 cells (ZR/EGFR) permits a response to EGF and can support proliferation independently of oestrogen [31]. Gene expression of ZR/EGFR cells stimulated with EGF for 7 days was directly compared with that of ZR-75-1 cells stimulated with oestrogen continuously. It is clear from this direct comparison that 247 genes are specifically altered more than 1.6-fold (Additional file 1). From the virtual experiment, which compares the EGF stimulation of ZR/EGFR cells with unstimulated ZR-75-1 cells, we can conclude that EGF modulates a large cohort of genes (707) at least 1.6-fold (Fig. 1). Statistical analysis of all 1006 genes shows a strong positive correlation for gene expression regulated by EGF and long-term oestrogen (*r*_s _= 0.67, *P *< 0.0001), but no significant association with genes induced early by oestrogen (*r*_s _= 0.16). As expected, *EGFR *is one of the most prominently changed genes in our analysis as a consequence of the transgene expression. In addition, the expression of genes implicated in signalling processes, cell adhesion and structure, protein modification, transport and metabolic processes is specifically regulated by treatment with EGF or shows a pronounced alteration in comparison with that in oestrogen-treated cultures (Fig. 1).

### Effects of overexpression of BCAR1 or BCAR3 in ZR-75-1 cells

We have previously shown that stable overexpression of BCAR1 or BCAR3 induces cell proliferation independently of oestrogen and anti-oestrogen [17,18]. In an attempt to pinpoint the effects of these cytoplasmic signalling molecules on global gene expression in cultures without added oestrogen, we compared BCAR3 and BCAR1, and BCAR3 and ZR-75-1, directly on microarrays, and calculated the gene expression relation between BCAR1 and ZR-75-1 as a virtual experiment. A total of 79 genes exhibited consistent differences in expression of at least 1.6-fold (Fig. 1). Few genes are modulated solely by the overexpression of a *BCAR *gene; most are also a target for hormonal and/or EGF stimulation (Fig. 1 and Additional file 1). As expected, the largest observed difference (up to 15-fold) in these comparisons was derived from the *BCAR3 *transgene expression. No cDNA sequence corresponding to the *BCAR1 *gene was present on this microarray.

Changes in gene expression specifically caused by BCAR3 overexpression in ZR-75-1 cells were seen for *CSTA*, *DLG7*, *FMOD*, *FOLR1*, *FOXJ1*, *HSPC242*, *IGFBP5*, *LGALS1*, *LIV1*, *NEDD4L*, *NELL2*, *PCDH7 *and *UBE2C*. In addition, a clear induction of several genes involved in glucose metabolism (*PGK1*, *LDHA*, *TPI1*, and moderate induction levels of *ALDOC*, *ENO1*, *ENO3 *and *PFKP*; Fig. 1) was observed. This coherent change in gene expression is unlikely to represent a culture artefact because no expression change was observed in these genes in BCAR3-transfected cells after 6 hours of induction with oestrogen (Additional file 1). Specifically altered genes in BCAR1-transfected ZR-75-1 cells include *APOD*, *ASS*, *CRIP1*, *ELL2*, *FOXM1*, *HSPG2*, *ID1*, *IL1R1*, *IL6ST*, *LGALS8*, *PDZK1*, *TK1*, *PPP3CA*, *TOMM20*, *VIPR1 *and various genes encoding ribosomal proteins (Fig. 1). A moderately opposing direction of gene expression change in these two transfectant cell lines compared with ZR-75-1 cells was indicated by *TFF3 *and *MGP *(Fig. 1). A set of genes (including *BF*, *BCL2*, *BIRC5*, *CDKN1A*, *INADL*, *KIAA0101*, *MAPKAPK2*, *MYB*, *P2RY10*, *PC4*, *PRCP*, *OCLN*, *RAB6KIFL *(= *KIF20A*), *SERPINA5*, *SLC7A2 *and *UGT2B11*) exhibited differential expression in both BCAR-transfected cell lines when compared with the parental cell line ZR-75-1.

### Verification of expression differences by quantitative PCR

To establish that the measured differences in gene expression on the microarrays did indeed reflect the concentration of the respective mRNAs, we performed quantitative RT–PCR (Q-PCR) on a selection of 22 genes on the same RNA samples and on additional RNA preparations from different culture conditions. Standard quantities of intact total RNA or mRNA were reverse transcribed and subjected to Q-PCR. To normalise the cDNA samples we used *HPRT1 *(not present on the microarray) as a reference. Results have been presented relative to non-stimulated ZR-75-1 cells to facilitate direct comparison with microarray data (Table 3). In general, we found good agreement between the levels of *HPRT1 *and another housekeeping gene (*HMBS*) in our experimental samples. The expression of *MYC *was fairly constant in our series, with the exception of a slight decrease in EGF-stimulated ZR/EGFR cells (Table 3). This is in agreement with the results of our microarray hybridisations, which showed *MYC *expression to be significantly reduced only by EGF (Fig. 1). Oestrogen targets such as *TFF1*, *PGR*, *PDZK1 *and *CTSD *are indeed increased by treatment of our ZR-75-1 cells and BCAR1-transfected and BCAR3-transfected cells with oestrogen (Table 3). These genes are already elevated after 6 hours of treatment with oestrogen (not by the pure oestrogen antagonist ICI 164,384), but their levels increase further (*TFF1 *up to 30-fold) after prolonged oestrogen treatment, in agreement with our microarray data. After stimulation of ZR/EGFR cells with EGF, the expression of *PGR *and *PDZK1 *was completely abolished, whereas *TFF1 *and *CTSD *levels were induced under EGF (Table 3). *TFF3 *and *MGP *levels were clearly induced after long-term treatment with oestrogen and reduced after stimulation with EGF. The levels of *ERα *(not present on the array) show some decrease after treatment of ZR-75-1-derived cell lines with oestrogen (Table 3). A much stronger decrease in *ER*α levels (10-fold) is achieved after 7 days of treatment of ZR/EGFR cells with EGF. *ERβ *levels were found to be reduced about 1000-fold compared with *ERα *in our cells and not strongly affected by the culture conditions. The levels of HER2/Neu (*ERBB2*) were decreased after treatment with both oestrogen and EGF. The expression levels of *BCAR3*, *EGFR *and *BCAR1 *were not strongly affected in the various cultures, except for the cells containing the introduced transgene. The observed expression modulation of *IGFBP5*, *IL1R1 *and *CSTA *in our microarray experiments is very well reproduced by Q-PCR (Fig. 1 and Table 3). Although oestrogen treatment causes a moderate decrease in these genes in ZR-75-1 cells, treatment of ZR/EGFR cells with EGF causes a marked effect (100-fold decrease in *IGFBP5 *after 7 days). The Q-PCR data also support the microarray data that BCAR3 cells have decreased levels of *IGFBP5 *mRNA and increased levels of *CSTA *mRNA in comparison with the BCAR1 and parental cells. *IL1R1 *and *APOD *levels are slightly modulated in BCAR1 cells, in agreement with the hybridisation data. *PSAT1 *levels were found to be further decreased in BCAR1 cells than suggested by the array experiments.

## Discussion

The expression of a large proportion of genes investigated on this cDNA microarray does not alter significantly after stimulation of ZR-75-1 cells with oestrogen, or ZR/EGFR cells with EGF, or transfection of *BCAR1 *or *BCAR3 *genes. Furthermore, different ZR-75-1-derived cell clones showed very similar expression profiles after growth manipulation with oestrogen, indicating the stability of the parental cell line and the absence of extensive variation between cell clones. This result is in agreement with previous observations that this human breast cancer cell line is extremely stable and is a suitable target for *in vitro *insertion mutagenesis with retrovirus [16]. The growth of this cell line is completely dependent on oestrogen, and the proliferation signal is mediated primarily through ERα because *ERβ *mRNA levels were very low. The *ERα *mRNA is readily detected in our cells by Q-PCR and is moderately decreased by oestrogen treatment (Table 3). In contrast, *ERα *mRNA is strongly decreased (about 10-fold) in EGF-treated ZR/EGFR cells, which might relate to the observation of growth interference between signalling by oestrogen and by EGF in these cells [31]. Of the 1006 significantly affected genes in our series of experiments, only few are immediate/early targets of oestrogen-activated ERα (Fig. 1 and Table 3). Most changes in gene expression observed in our cell model are the result of long-term culture with either oestrogen or EGF. Many genes here identified as oestrogen targets have previously been reported to be directly regulated by oestrogen using conventional northern blotting, serial analysis of gene expression (SAGE) [35] or gene expression profiling [36-40] (see also Fig. 1, 'cell lines' column). Good concordance with literature data was observed for the immediate and late targets of oestrogen in our cells (categorised data, *r*_s _= 0.42 and 0.41, respectively; *P *< 0.01). As expected, no association between EGF-regulated gene expression and reported oestrogen targets was seen (categorised data, *r*_s _= - 0.07). Undisputed early targets are *TFF1*, *CTSD*, *CCND1*, *PGR*, *PDZK1 *and *MYB*, whereas induction of *IGFBP4 *by oestrogen was reported to be dependent on the presence of serum in MCF7 cells [37]. Mostly overlapping results for oestrogen-regulated genes in MCF7 cells have been presented recently [40]. Differences between the various cell line models might explain individual differences (see Fig. 1, for example *MYC*, *XBP1 *and *MGP*). *STC1 *is rapidly induced in our experiments but has been reported not to be regulated in MCF-7 cells with the use of SAGE and microarrays [35,40]. In contrast, its family member *STC2 *was strongly increased by oestrogen treatment of MCF7 cells [35,37]. On our microarrays we did observe a moderate induction (1.7-fold) in *STC2 *transcript levels after induction with oestrogen for 6 hours, essentially parallel to *STC1 *(Fig. 1).

Linking expression databases derived from cell line models and clinical samples provides the opportunity to extract additional information. We have connected our *in vitro *gene expression data with the public results of gene expression profiling of clinical breast cancer specimens [28,41] through the Unigene cluster number. About one-fifth of the 1006 genes in our selection were reported to be associated with ER status, *BRCA *mutation status and/or breast cancer prognosis (Fig. 1). Comparison of the categorised data of early oestrogen-regulated gene expression and expression association with ER status reveals a positive correlation (*r*_s _= 0.21, *P *< 0.002). Clear examples of genes showing a positive correlation with ER status in breast carcinoma and oestrogen-induced gene expression in ZR-75-1 cells are *CCND1*, *GFRA1*, *GJA1*, *IGFBP4*, *IL6ST*, *MEIS3*, *MYB*, *PDZK1*, *PGR*, *SERPINA5 *and *TFF1 *(Fig. 1). Examination of the genes regulated in our cells by long-term treatment with oestrogen reveals an unexpected inverse relation (*r*_s _= - 0.23; *P *< 0.002) with expression association to ER status of the tumour. About half of the genes regulated by long-term oestrogen and not regulated by EGF exhibit concordance with ER status, whereas most of the genes regulated similarly by both treatments show a discordant relation with ER expression (Fig. 1). A much stronger negative relation exists between EGF-regulated gene expression and expression association with ER status (*r*_s _= - 0.43; *P *< 0.0001), which concurs with the established inverse relation between ER and EGFR in breast cancer [7,8]. The results of this comparison of cell line expression data with profiles of clinical samples indicate that part of the molecular markers for ER status in primary breast tumours might indeed represent genuine ER targets. Various other markers are not linked to oestrogen action but might reflect activation of the EGFR pathway in ER-negative tumour cells. Partial overlap was also reported for genes associated with breast cancer ER status and oestrogen-responsive genes in MCF7 cells [40]. Some genes reported to be associated with the prognosis of node-negative breast cancer (*TK1*, *FBP1 *and *IGFBP5*) or with *BRCA *mutation-induced disease (*CPE *and *P2RY10*) seem to be regulated by BCAR1 and/or BCAR3 (see Fig. 1). In addition, the expression of *IL1R1*, *FOXM1 *and *PC4 *changes markedly during the progression of normal prostate to metastasised prostate cancer [27]. These observed relations of genes regulated by BCAR1 and/or BCAR3 with clinical features of malignancies remain interesting and are targets for further study.

The overall results show that BCAR1-transfected or BCAR3-transfected cells in unsupplemented cultures exhibit only modest changes in gene expression compared with unstimulated ZR-75-1 cells (Fig. 1 and Table 3). The prominent changes in gene expression induced by BCAR3 are upregulation of *CSTA*, *HSPC242 *and *LGALS1 *and downregulation of *IGFBP5*, *NEDD4L *and *PCDH7*. These genes are involved in protein degradation, cell–cell adhesion, the assembly of extracellular matrix and control of cell growth and metabolism. In BCAR1-transfected cells, upregulation of *BIRC5*, *ELL2*, *FOXM1*, *ID1*, *IL1R1*, *MYB *and *TK1 *and downregulation of *APOD*, *IL6ST *and *LGALS8 *was observed. Several of these genes modulated by BCAR1 have been shown to be important in cell signalling and in the regulation of cell proliferation or possibly in increasing cell survival. These clearly different patterns of gene expression in BCAR1 and BCAR3 transfectants indicate that signalling proceeds along alternative pathways. This contrasts with the co-occurrence of BCAR1 and BCAR3 in a protein complex in some of our cell models (Ton van Agthoven, Arend Brinkman, Lambert CJ Dorssers, unpublished results) and the functional association of BCAR1/p130Cas and BCAR3/AND-34 in cell migration [42]. From the profiles in Fig. 1 and Additional file 1 it is clear that partial overlap exists in the expression profiles of the BCAR1 and BCAR3 transfectants with both oestrogen-induced and EGF-induced cells. Most of these genes are modulated in most experiments and thus might represent expression features of proliferating ZR-75-1 cells. The remaining overlap with either oestrogen-modulated or EGF-modulated genes is limited, making it highly unlikely that the *BCAR *genes use major parts of these signalling pathways. This observation agrees with previous results showing that BCAR1 and BCAR3 cell lines generated by retroviral insertion mutagenesis had all lost ERα protein expression and did not acquire responsiveness to EGF [16,17]. Furthermore, growth of BCAR1 and BCAR3 transfectants (which are fully responsive to oestrogen; Table 3 and Additional file 1) was not stimulated by anti-oestrogen [17,18], suggesting that there is no role for ERα in the anti-oestrogen-resistant proliferation of these cell models. In clinical specimens, BCAR1 was found to be an independent marker (multivariate analyses also including ERα) for early recurrence of breast cancer and for failure of tamoxifen treatment of recurrent disease [21-24]. Because not all genes are present on this microarray and only a limited set of experimental conditions have currently been analysed in our cell model, we cannot exclude from these microarray experiments the possibility that the BCAR transfectants selectively use components of the hormonal receptor and/or growth factor receptor signalling pathways for proliferation control. In addition, growth control mediated by BCAR1 and BCAR3 might not be reflected in gene expression but could also be supervised at the level of regulatory protein activation.

Our results present an overview of gene expression changes after perturbation of ZR-75-1 breast cancer cells with treatment with oestrogen or EGF or by the overexpression of *BCAR *genes. The data suggest that oestrogen-independent cell proliferation induced by overexpression of BCAR1 or BCAR3 does not depend merely on the oestrogen-signalling or EGFR-signalling pathways. Because BCAR1 protein levels have been associated with clinical outcome for breast cancer patients, further studies are needed to resolve the underlying mechanism. This study also shows that important cell biological properties such as oestrogen-independent proliferation can be regulated in several subtle ways and thus might be hidden in the excess of gene expression differences observed in profiles of specimens from patients. The combination of expression profiles of relevant cell models, which can be manipulated *in vitro*, and high-quality specimens from patients might permit the identification and understanding of the important cellular pathways contributing to major clinical features of malignant diseases [43]. This information could ultimately lead to the development of improved or novel treatment strategies for breast cancer.

## Abbreviations

BCAR = Breast Cancer Anti-oestrogen Resistance; DE = differential expression; E2 = oestradiol; EGF = epidermal growth factor; EGFR = epidermal growth factor receptor; ER = oestrogen receptor; P130Cas = Crk-associated substrate; Q-PCR = quantitative RT–PCR; R/BCS = RPMI 1640 medium supplemented with 10% bovine calf serum; *r*_s _= Spearman rank correlation; RT–PCR = reverse transcriptase-mediated polymerase chain reaction; SAGE = serial analysis of gene expression; ZR/EGFR = EGFR-transfectant cell line ZR/HERc(1A).

## Competing interests

The author(s) declare that they have no competing interests.

## Supplementary Material

Additional File 1 A TIFF file showing hierarchical clustering of gene ratios. Signal intensity tables were imported into Rosetta Resolver, combined for dye swapping and evaluated using the Incyte/UniGEM error model. A total of 2373 genes displaying at least once a *P *value of 0.01 or less were used for hierarchical clustering. In this colour picture, increased expression is shown in red and decreased expression is shown in green. Black represents no change and grey indicates missing data. The compared RNA samples are indicated. The gene names and log(ratios) are provided in Additional file 2. Click here for file

Additional File 2 An Excel file containing gene expression data, namely Incyte spot ID, accession number, sequence name and description, and the Rosetta Resolver output data columns: log(ratio), *P *value and log(error). Click here for file

Additional File 3 An Excel file containing all data from the actual and virtual experiments meeting the selection criteria (see Fig. 1), Unigene cluster number, gene name and description, and cited literature data with regard to oestrogen-regulated gene expression in cell lines and associations with tumour phenotypes. Ordering is in accordance with the hierarchical clustering of all genes, using the columns depicted in Fig. 1. Click here for file

## Figures and Tables

**Table 1:** Hybridisation experiments

RNA no.	Microarray hybridisations							
1	BCAR3 (R/BCS)	BCAR3 (E2 6 h)	BCAR3 (R/BCS)	ZR-75-1 (E2 6 h)	ZR-75-1 (E2 7 d)	ZR-75-1 (E2 7 d)	ZR-75-1 (E2 7 d)	ZR-75-1 (E2 7 d)
2	BCAR1 (R/BCS)	BCAR3 (R/BCS)	ZR-75-1 (R/BCS)	ZR-75-1 (R/BCS)	ZR-75-1 (R/BCS)	BCAR1 (R/BCS)	BCAR3 (R/BCS)	ZR/EGFR (EGF 7 d)

RNA samples were prepared from cell lines pretreated for 5 days in RPMI 1640 medium supplemented with 10% bovine calf serum (R/BCS) and subsequently cultured for the indicated durations in R/BCS medium supplemented with oestrogen (E2), epidermal growth factor (EGF) or R/BCS medium without further additions. All hybridisations were performed in duplicate with swapping of the dyes.

**Table 2:** Primer information

Gene		Sequence 5'→3'
*HPRT1*	F	TATTGTAATGACCAGTCAACAG
	R	GGTCCTTTTCACCAGCAAG
*HMBS *(= *PBGD*)	F	CATGTCTGGTAACGGCAATG
	R	GTACGAGGCTTTCAATGTTG
*IGFBP5*	F	GGGTTTGCCTCAACGAAAAG
	R	TTTCTGCGGTCCTTCTTCAC
*ESR1 *(*ERα*)	F	GAGCACCCAGGGAAGCTAC
	R	CATCAGGTGGATCAAAGTGTC
*CSTD *(cathepsin D)	F	CACGGGCTCCTCCAACCT
	R	GGACTTGTCGCTGTTGTACTTGTG
*TFF1 *(PS2)	F	ATGGCCACCATGGAGAAC
	R	TTCACACTCCTCTTCTGG
*MYC*	F	GAGCCCCTGGTGCTCCAT
	R	CGATTTCTTCCTCATCTTCTTGTTC
*ESR2 *(*ERβ*)	F	TCAGCCTGTTCGACCAAGTG
	R	GGCCTTGACACAGAGATATTC
*PGR*	F	CAAGTTAGCCAAGAAGAGTTC
	R	ACTTCGTAGCCCTTCCAAAG
*ERBB2 (HER2/Neu*)	F	GTCTACAAGGGCATCTGGAT
	R	GTGGATGTCAGGCAGATGC
*BCAR3*	F	GCGGTGGAACTGAAGGATTC
	R	TGGCAGTTTGGGTGTACTGG
*BCAR1*	F	CTGCCCAGGATATTTACCAG
	R	CGTCATACACCTCCAGCAAC
*EGFR*	F	CGGGACATAGTCAGCAGTG
	R	GCTGGGCACAGATGATTTTG

Gene	Product code	

*CDKN1A*	HS00355782_m1	
*PGK1*	Hs99999906_m1	
*IL1R1*	Hs00168392_m1	
*CSTA*	Hs00193257_m1	
*TFF3*	Hs00173625_m1	
*FMOD*	Hs00157619_m1	
*PDZK1*	Hs00420042_m1	
*APOD*	Hs00155794_m1	
*MGP*	Hs00179899_m1	
*PSAT1*	Hs00253548_m1	

F, forward; R, reverse. The gene names are defined in Additional file 3.

**Table 3:** Relative gene expression in ZR-75-1-derived cell lines

	ZR-75-1 cells						BCAR1-transfected cells				BCAR3-transfected cells				EGFR-transfected cells		
	
Addition...^a^	None	ICI	Oestrogen				None	Oestrogen			None	Oestrogen			None		EGF
		6 h	6 h	30 h	96 h	Cont.		6 h	30 h	96 h		6 h	30 h	96 h		3 days	7 days
Gene	3 (4)^b^	1 (1)	2 (4)	1 (1)	1 (3)	1 (3)	2 (6)	2 (3)	1 (1)	1 (1)	2 (4)	2 (3)	1 (1)	1 (1)	1 (3)	1 (2)	1 (4)
*HMBS*	(23.1) 1^c^	0.58	0.79	0.42	0.49	1.22	0.93	0.75	0.51	0.93	0.59	0.56	0.41	0.64	0.93	0.80	0.99
*FMOD*^d^	(29.4) 1	1.60	1.47	0.79	1.04	0.95	0.58	0.83	0.71	0.81	0.95	1.09	1.00	1.59	1.02	0.94	0.54
*PGK1*^d^	(22.3) 1	1.33	1.02	1.08	1.18	1.18	1.41	0.94	1.07	1.61	1.46	2.10	0.97	2.11	0.70	2.23	0.94
*CDKN1A*^d^	(25.8) 1	0.60	0.72	0.47	0.66	2.03	1.45	1.24	0.61	1.34	1.19	1.37	0.81	1.78	1.26	2.11	3.60
*MYC*	(23.3) 1	0.55	1.21	0.41	0.90	0.97	0.72	0.98	0.47	0.74	0.47	0.60	0.51	0.76	1.00	0.44	0.26
*CTSD*	(18.7) 1	0.68	2.13	2.25	1.94	3.95	0.91	1.89	1.87	3.02	0.77	1.43	1.90	4.21	1.78	2.38	1.95
*TFF1*	(21.2) 1	0.56	2.07	9.28	20.45	36.67	0.99	2.38	13.37	32.60	0.94	1.35	9.30	21.37	4.71	5.77	2.64
*PGR*	(24.6) 1	0.71	9.08	9.24	12.00	7.52	0.83	7.24	9.29	7.65	0.38	2.22	3.55	5.62	0.56	0.32	0.05
*PDKZ1*^d^	(26.4) 1	0.92	5.26	14.75	10.12	16.66	0.64	1.85	11.86	11.81	0.52	1.93	7.26	9.10	1.17	0.08	0.03
*TFF3*^d^	(23.0) 1	0.50	1.03	2.64	6.19	11.56	0.84	1.30	2.60	7.29	1.21	1.23	2.22	4.02	1.09	0.79	0.10
*MGP*^d^	(22.8) 1	0.83	1.09	1.54	2.99	1.34	0.93	1.00	3.12	3.15	0.89	0.78	2.14	1.11	0.42	0.86	0.23
*IGFBP5*	(17.6) 1	0.80	0.92	0.24	0.45	0.17	0.91	0.74	0.27	0.49	0.33	0.30	0.22	0.28	1.46	0.04	0.01
*APOD*^d^	(21.5) 1	0.96	0.89	0.53	0.25	0.09	0.37	0.36	0.18	0.08	0.60	0.59	0.36	0.27	0.32	0.02	0.04
*CSTA*^d^	(27.7) 1	0.57	0.86	0.08	0.06	0.06	0.74	0.53	0.08	0.09	2.95	2.86	0.88	0.44	0.04	0.01	0.02
*HER2/Neu*	(20.4) 1	0.77	0.75	0.18	0.29	0.39	0.73	0.48	0.20	0.25	0.68	0.53	0.29	0.37	1.16	0.51	0.49
*IL1R1*^d^	(27.5) 1	0.76	0.50	0.10	0.09	0.04	1.54	0.54	0.07	0.15	0.64	0.40	0.14	0.23	0.84	0.12	0.21
*ERα*	(20.2) 1	0.54	0.86	0.36	0.41	0.27	0.50	0.55	0.31	0.29	0.35	0.39	0.28	0.33	0.79	0.12	0.07
*ERβ*	(32.7) 1	1.21	1.93	0.05	1.57	1.62	1.61	1.40	0.63	0.60	0.84	1.78	0.42	3.19	1.58	1.08	1.89
*BCAR3*	(24.2) 1	0.74	0.51	0.40	0.46	0.45	0.64	0.25	0.14	0.23	18.30	14.36	13.19	24.78	0.35	0.60	0.69
*BCAR1*	(24.8) 1	0.93	1.17	0.71	0.69	1.31	55.96	31.90	13.67	22.12	0.80	1.01	0.58	0.36	1.01	1.13	1.07
*PSAT1*^d^	(35.6) 1	0.86	0.67	0.40	0.97	2.64	<0.01	<0.01	<0.01	<0.01	0.87	1.16	0.84	0.76	1.65	3.36	3.64
*EGFR*	(35.0) 1	0.25	0.84	1.62	1.75	1.27	1.47	2.56	2.31	1.25	2.24	2.15	2.75	4.13	8126	11045	10582

All expression levels have been normalised for HPRT1 levels and are presented relative to non-stimulated ZR-75-1 cells. The gene names are defined n Additional file 3.

^a^The nature and duration of the culture additions (ICI, oestrogen antagonist ICI 164,384).

^b^The number of biological replicates and total number of analyses (in parentheses).

^c^Gene expression level was set at 1.0 for non-stimulated ZR-75-1 cells and the detection threshold cycle (C_t_) value for each gene is shown in parentheses (23 for HPRT1).

^d^Quantitative polymerase chain reaction with assay on demand; others with SYBR green.
