# Supplementary figures and images for: Breast cancer oestrogen independence mediated by BCAR1 or BCAR3 genes is transmitted through mechanisms distinct from the oestrogen receptor signalling pathway or the epidermal growth factor receptor signalling pathway
Source: Breast Cancer Res. 2004 Nov 16;7(1):R82–92. doi: 10.1186/bcr954 (PMC1064102; doi:10.1186/bcr954)

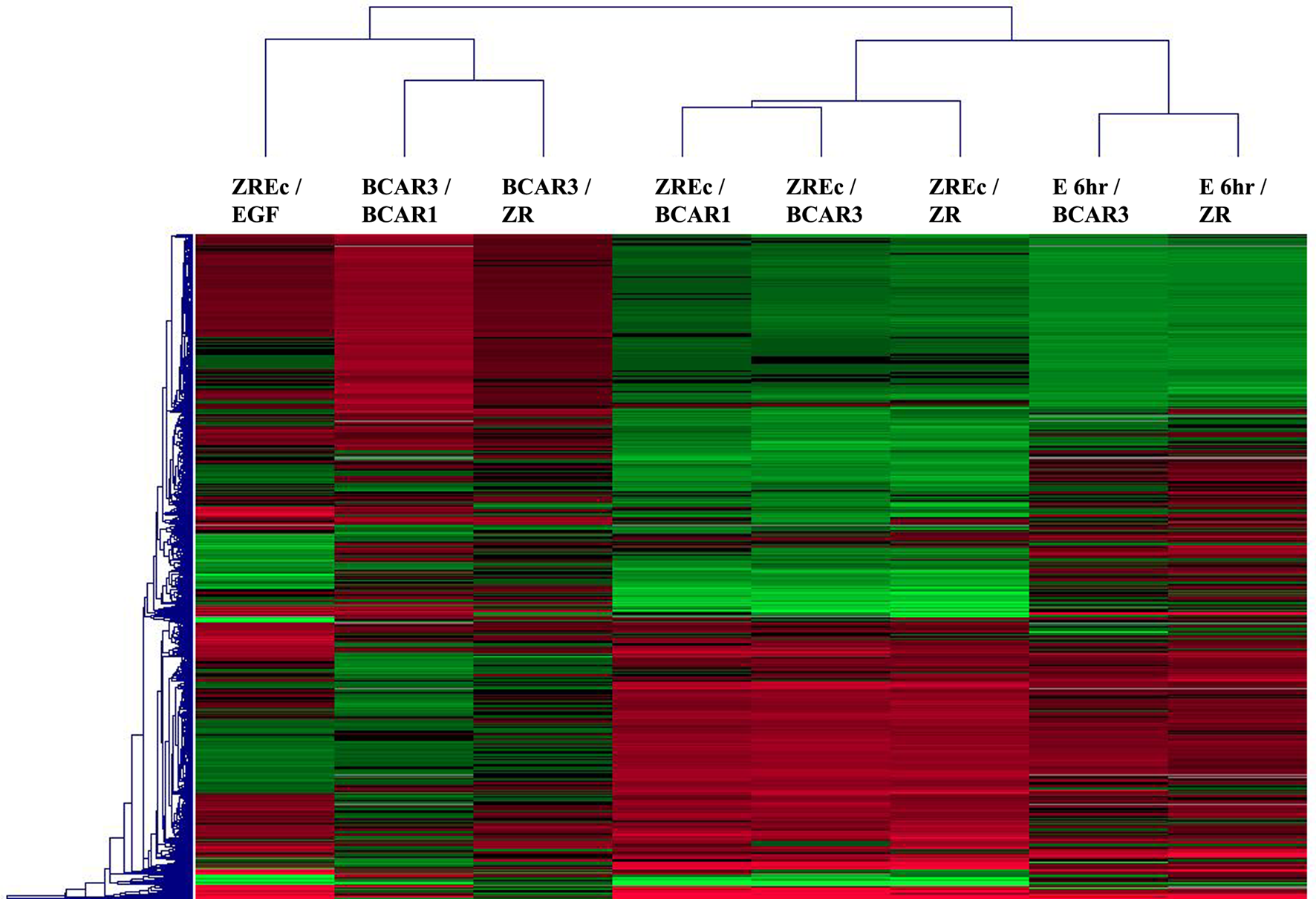

Supplement: Additional File 1 — A TIFF file showing hierarchical clustering of gene ratios. Signal intensity tables were imported into Rosetta Resolver, combined for dye swapping and evaluated using the Incyte/UniGEM error model. A total of 2373 genes displaying at least once a P value of 0.01 or less were used for hierarchical clustering. In this colour picture, increased expression is shown in red and decreased expression is shown in green. Black represents no change and grey indicates missing data. The compared RNA samples are indicated. The gene names and log(ratios) are provided in Additional file 2. [file bcr954-S1.tiff]
